# Spina Bifida, Treated by Iodine—Cure by One Injection

**Published:** 1861

**Authors:** Daniel Brainard

**Affiliations:** Professor of Surgery in Rush Medical College, Etc.


### SELECTED.

### SPINA BIFIDA, TREATED BY IODINE—CURE BY ONE INJECTION.

By DANIEL BRAINARD, M.D.,
*Professor of Surgery in Rush Medical College, etc.*

*November 7th*, 1860, a girl three years old was brought to me to be treated for spina bifida. The child was intelligent, healthy, and well formed in every respect excepting the tumor situated over the sacrum. This was eight inches in circumference at the base, about two and a half inches in height, conical, translucent, elastic, and covered with healthy skin excepting a small point at the lower part where it was discoloured like the vestige of a nævus. Below the tumor there was an umbilicated depression like a cicatrix adhering to the sacrum.

*Operation.*—Nov. 10th, 1860, assisted by Prof. Ephraim Ingalls and Dr. Edwin Powell, the operation was performed as follows: A small sized hydrocele trocar was carried into the tumour at its base on the right side, and six ounces of fluid drawn off; while this was flowing, pressure was made by an assistant, and as the sac was emptied, the pulp of the thumb was pressed upon and partly into the opening in the spine which it exactly filled, so as to close it as perfectly as possible. Half an ounce of a solution (five grains iodine, fifteen grain iodide potass to the ounce distilled water) at the temperature of the body, was then injected through the canula and after a few seconds allowed to flow out; distilled water at the temperature of the body was thrown in to wash out the iodine, and two ounces of the fluid first drawn from the sac and kept at the same temperature, were re-injected and the canula withdrawn. From movements of the child, some bubbles of air passed into the sac, and as these could not readily be brought out they were left.

During the operation the child was kept under the influence of chloroform, of which it required a very unusual quantity, and, when this was finished, it remained fifteen minutes in a quiet sleep.

The puncture was dressed with a strip of isinglass plaster and a compress supported by a band around the pelvis placed over it.

On awakening, the child made efforts to vomit and seemed to be severely nauseated for half an hour, when it fell into a light sleep. During the afternoon it vomited occasionally, refused food, asked for cold water, and urinated often.

11*th*. Has been restless during the night, probably from being kept lying on the face; pulse and heat of skin natural, puncture at eleven o'clock; twenty-four hours after the operation, found to be leaking; tumor tense; applied more perfect compression over it. During the day child drank freely of toast water, and in the afternoon fell into a free warm perspiration, which lasted two hours.

12*th*. Has slept well, asked for toasted bread twice, and ate it; seems perfectly well.

13*th*. Tumour tense, redness around the puncture; applied cloths dipped in warm water.

15*th*. Puncture leaking; passed a fine needle through the edges, tied a fine thread around it, in form of twisted suture.

17*th*. Tumour tense; tapped it on the left side near the base on sound skin with an exploring trocar, and drew off six ounces of slightly turbid fluid. Continued warm water applications.

19*th*. First puncture leaks slightly; needle withdrawn and compress supported by a truss placed over it.

20*th*. First puncture closed; child in its usual health, tumour flaccid, walls slightly firmer than before the operation. *Translucency quite gone.* Applied an India-rubber band around the pelvis so as to compress the sac.

25*th*. Tumour much diminished; walls firmer. Removed the band and substituted an umbilical truss, the pad of which was placed over the sac.

30*th*. Child in good health; tumour diminishing.

*Dec.* 3. Tumour but imperfectly fluctuating and evidently filled with semi-solid contents.

From this time to December 31st the truss was kept applied with compresses of fine linen within the centre, so as to press the skin into the opening in the spine. It was taken off and replaced daily, so as to avoid excoriation. The child suffered no pain, was in perfect health, and played about as before the operation.

31*st*. The skin at the centre of the tumour is adherent to the opening in the spine, which is felt to be closed. A little fulness around the base at the upper part alone marks the vestige of the tumour. I advised the continued wearing of the truss unless it should excoriate, and the parents left for home.

*Feb.* 10. Child has remained well; no tendency to return in the tumour. Truss has been left off for several weeks at a time when the pressure produced restlessness.

The fluid first drawn from this tumour was perfectly limpid, had the peculiar odour of the cerebro-spinal fluid, was very slightly albuminous.

With the microscope, only a few epithelial scales, and a trace of coagulated fibrin, could be detected. That drawn on the seventh day was turbid, and on cooling deposited a sediment composed of coagulated albumen and fibrin, with what appeared to me to be pus-globules here and there. These, if I was not mistaken in their character, must have come from the internal orifice of the puncture, which was still leaking.

*Remarks.*—This is the seventh case of spina bifida which I have treated by iodine injections. In no case have I seen it produce dangerous symptoms. It is the third unaccompanied by hydrocephalus; all these three has been perfectly and permanently cured—one with thirteen injections, one with two, and the last with one. In the last two, means were taken to prevent the passage of the solution into the spinal canal. In one, the tumour being pediculated, this was easily done; in the other, the means above described were resorted to with satisfactory results. When this can be effected, the solution may be used strong, and washed out so as to render one or two operations sufficient. The object of reinjecting some of the fluid in the case above reported, was to enable a band around the pelvis to effects some pressure on the cord.

The operation is so delicate that it is not easy in any case to fulfil all the requisite conditions. Thus, in the above case, the walls were so thin at the point of puncture, that it did not close for ten days, constituting a source of danger. Some bubbles of air also passed in, too which too much consequence need not be attached, as no harm resulted; but it would be preferable to avoid such an occurrence.

Applications of collodion for the cure of spina bifida have been recently suggested. When the walls are thick and firm this may be safe, and will be as serviceable as other forms of compression. When the covering is thin, it is dangerous. Dr. James Gow reported, in the *Chicago Medical Journal* for November, 1860, a case where it caused ulceration and rupture. Prof. Gross reports, in the *North American Medico-Chirurgical Review* for November, 1860, a case of this malformation, treated by injection of iodine. "The tumour was thoroughly painted over with collodion." The tumour opened (not at the point of puncture) on the second day, and on the sixth it "burst completely." Although the covering is stated to have been "on the point of bursting" before the operation, it seems probable that the collodion hastened if it did not cause the rupture.

In another case by Prof. Gross (*ib.*), where about ten injections were used in eight weeks, the collodion was kept applied, and the tumour burst, causing death. Concerning this case, there are two points deserving notice :—

1. As there is no reason to suppose that the rupture was caused by the injections, it must be attributed to the collodion.

2. As the tumour was found on dissection to be "about one-third obliterated by coagulable lymph," the inquiry naturally suggests itself, whether one or two injections would not have been sufficient. A certain thickening of the walls has indicated to me that further injections were unnecessary, and the loss of translucency, or the turbid appearance of the fluid withdrawn are indications of a change in the structure and action of the lining membrane, sufficient, with judicious pressure, to effect a cure.

As these cases of Prof. Gross, taken without detail, are calculated to discourage the trial, it may be well to note that, although about ten injections were made so as to pass in some degree into the spinal canal, "the child early suffered from the convulsions after the operations, and they always readily yielded to a dose of caster oil."

I regard both the cases of Prof. Gross as tending to show the safety and efficiency of this method of treatment, as in both plastic lymph was deposited within the sac without any dangerous symptoms attributed to the operation having occurred.

The manner in which collodion acts in producing ulceration is threefold : 1. By vesication; 2. By expelling the blood from the thin walls; 3. By increasing the tension of the walls of the sac, which it does by diminishing its size.

